# EMPLOYMENT AND FINANCIAL STATUS BEFORE AND AFTER THE COVID-19 PANDEMIC AMONG OLDER PEOPLE IN ENGLAND

**DOI:** 10.1093/geroni/igad104.1379

**Published:** 2023-12-21

**Authors:** Jingmin Zhu

**Affiliations:** University College London, London, England, United Kingdom

## Abstract

The COVID-19 pandemic has negatively affected society and the economy worldwide. By the end of 2021, most legal COVID-19 restrictions were lifted in England, pushing society into a post-pandemic phase. However, it remains unclear whether the post-pandemic world has convalesced, particularly with regard to socioeconomic circumstances among older people. According to the English Longitudinal Study of Ageing (ELSA), the percentage of employment among older people aged 50 years and above living in England decreased dramatically from 24.9% in 2018/9 to 17.6% in June/July 2020 and 19.8% in November/December 2020. Preliminary results from data collected between October 2021 and November 2022 suggest that post-pandemic the percentage of older people in paid work bounced back to pre-pandemic levels. Nevertheless, the post-pandemic financial status was worse than that before the pandemic. Whereas approximately 22% of respondents were worried about the future financial situation during the pandemic, this prevalence increased to nearly 30% in 2021/2. Moreover, there is an indication that some groups (including those with low education; those in routine and manual occupations; and those living alone) fared worse in the post-pandemic times. Overall, despite some evidence suggesting that the employment of English older people has bounced back to pre-COVID-19 pandemic levels, their financial situation seems to have deteriorated, most likely reflecting also the current rising cost of living and financial crisis in the UK.

